# Assessment of Common Anaesthetic and Clinical Indices of Multimodal Therapy of Propofol, Xylazine, and Ketamine in Total Intravenous Anaesthesia in West African Dwarf Goat

**DOI:** 10.1155/2014/962560

**Published:** 2014-10-16

**Authors:** Ukwueze Celestine Okwudili, Eze Chinedu Athanasius, Udegbunam Rita Ijeoma

**Affiliations:** ^1^Department of Veterinary Surgery and Theriogenology, College of Veterinary Medicine, Michael Okpara University of Agriculture, PMB 7267, Umudike, Umuahia, Abia State, Nigeria; ^2^Department of Veterinary Surgery and Radiology, Faculty of Veterinary Medicine, University of Nigeria, Nsukka, Nigeria

## Abstract

The assessment of anaesthetic and clinical indices of multimodal therapy of propofol, xylazine, and ketamine was done in West African Dwarf (WAD) goat. Sixteen healthy male WAD goats were assigned into four treatment groups, namely, control (group A) (ketamine 5 mg/kg + xylazine 0.05 mg/kg), group B (propofol 5 mg/kg + xylazine 0.05 mg/kg), group C (propofol 5 mg/kg + ketamine 5 mg/kg), and group D (propofol 2.5 mg/kg + ketamine 2.5 mg/kg + xylazine 0.05 mg/kg). All drugs were administered intravenously. The multimodal therapy decreased significantly (*P* < 0.05) the heart rate in groups A, B, and D. Also respiratory rate significantly (*P* < 0.05) decreased in groups A, B, and D but significantly (*P* < 0.05) increased at 20 min after induction in group C. However, temperature significantly (*P* < 0.05) decreased in groups A, B, and C. The induction was good and smooth in groups B and D. Surgical anaesthetic time was longer in groups B and D and shorter in group C. The quality of recovery was good in groups B and D. Side effects such as salivation and apnoea were observed in all groups. In conclusion, the multimodal therapy could be used successfully. However, group D could be the best combination considering the parameters measured.

## 1. Introduction

Anaesthesia is an indispensible prerequisite for most surgical interventions both in humans and in animals [1]. It is supposed to provide reversible unconsciousness, amnesia/analgesia, muscle relaxation, and immobility with minimal adverse effects, rapid and smooth recovery of protective reflex and psychomotor function [2, 3]. This can be possible through the use of balanced anaesthetic technique as no single drug provides all the components of general anaesthesia. The balanced anaesthetic technique involves combining two or more anaesthetic drugs to achieve the targeted components of general anaesthesia while minimizing the negative effects of individual drugs on cardiopulmonary function [4].

Ketamine produces stable haemodynamics during anaesthesia as a result of its stimulatory effect on the sympathetic nervous system, which counteracts the depressant effects of other drugs used during anaesthesia [5]. It can be used for anesthesia in sheep and goat without the fear of causing convulsion [6]. Muscle relaxation is poor, but it can be improved by sedatives such as diazepam or xylazine [7]. Ketamine and propofol multimodal therapy allows a reduction in the hypnotic dose of propofol and a decrease in the cardiovascular depression induced by this drug [8].

Propofol (2,6-diisopropylphenol) is a phenolic compound unrelated to any other general anesthetics. It is a nonbarbiturate, nondissociative, and noncumulative intravenous anesthetic agent [7]. It has good quality anesthesia, rapid onset, and short duration of action, with rapid recoveries making the drug potentially useful in ruminants, in which these features are particularly desirable [9]. It is used alone and in combination with other drugs in dogs, cats, cattle, horse, ponies, and goats [4, 5, 7, 10, 11]. Reports on the use of propofol for induction and maintenance of anaesthesia have indicated its suitability in goats [7, 9, 12]. Propofol has been shown to be compatible with wide range of drugs used for premedication, inhalation anesthesia, and neuromuscular block [7]. Since it has little or no analgesic property, it is recommended to combine propofol with an analgesic agent (Opoid or alpha-2-adenoreceptor agonist) for painful procedures.

Xylazine is an alpha-2-adenoreceptor agonist, is nonopioid group with analgesic, sedative, and muscle relaxant effects, and is used commonly in veterinary practice [6]. Xylazine is a commonly used sedative in ruminants but there are concerns about the threat of hypoxaemia associated with its use in small ruminants [13]. It has been used in combination with propofol in sheep [12] and with ketamine in intravenous general anaesthesia in goat [14].

Continuous search for proper drug multimodal therapy that will offer good quality anaesthesia, analgesia, and fast recovery with mild or no side effect using drugs of known anaesthetic quality such as xylazine, ketamine, and propofol is highly imperative.

The objective of this work was to evaluate the changes in common indices in multimodal therapy anaesthetic of propofol, ketamine, and xylazine in total intravenous anaesthesia in West African Dwarf (WAD) goats.

## 2. Materials and Methods 

### 2.1. Drugs

The drugs used were ketamine 50 mg/mL injection (Laborate Pharmaceuticals, India), propofol 10 mg/mL injection, 20 mL vial (Popular infusion Ltd., Tongi Bangladesh), and xylazine 20 mg/mL injection (Kepro Ltd., Holland).

### 2.2. Experimental Animal

Sixteen healthy male WAD goats of average weight of 15 kg were used for the study. They were randomly assigned into four groups (A–D) of four goats each. The goats were fed* ad libitum* with grasses and water throughout the experimental period.

### 2.3. Anaesthetic Effect

Control (group A) goats were anaesthetized using xylazine (0.05 mg/kg body weight) and ketamine (5 mg/kg body weight) multimodal combination. Group B goats were anaesthetized using a combination of xylazine (0.05 mg/kg body weight) and propofol (5 mg/kg body weight). Group C goats were anaesthetized using ketamine (5 mg/kg body weight) and propofol (5 mg/kg body weight), while, in group D, xylazine (0.05 mg/kg body weight), ketamine (2.5 mg/kg body weight), and propofol (2.5 mg/kg body weight) were administered simultaneously. All drugs were administered intravenously through jugular vein.

### 2.4. Anaesthetic Maintenance

This was done immediately after return of pain reflexes. In the control (group A) animals, anaesthesia was maintained at 25.00 ± 5.00 min and 40.00 ± 2.00 min after induction with a total of 3 mls of ketamine (5 mg/kg body weight). Animals in group B were given repeated injection of propofol 5 mg/kg body weight at 22.00 ± 3.00 min and at 39.00 ± 2.00 min after induction with a total of 10 mls. In the group C, anaesthesia was maintained at 18.00 ± 2.00 min and at 35.00 ± 3.00 min after induction with propofol 5 mg/kg body weight with a total of 10 mls. Also anaesthesia was maintained in group D at 30.00 ± 4.00 min and 50.00 ± 3.00 min with propofol 5 mg/kg body weight with a total of 15 mls titrated to effect.

### 2.5. Physiological Variable

The heart rate (Hr), respiratory rate (Rr), and rectal temperature (rt°) were measured at 0 min before treatment to ascertain the baseline values and subsequently every ten (10) min interval following the induction till the recovery of each animal.

### 2.6. Anaesthetic Indices

A modified method of determination of quality of anaesthesia according to Adetunji et al. [15] was used to obtain the anaesthetic indices.

#### 2.6.1. Onset of Flank Analgesia

This was done by recording the time (in min) of the initial injections of the drugs (*t*_0_) and the time of disappearance of flank twitch reflex (*t*_*z*_). The difference between the former and the later gave the time of the onset of flank analgesia.

#### 2.6.2. Surgical Anaesthetic Time

This was done by noting the time of disappearance of flank twitch reflex (TD) following the intravenous injection and the time of reappearance of flank twitch. Then the difference between the time of disappearance (TD) and the time of reappearance (TA) of flank twitch reflex gave the duration of flank analgesia time (DA): DA = TA − TD.

#### 2.6.3. Duration of Recumbency

This was measured by recording the time when anaesthesia induced recumbency in the goat and then the time when the goat was able to maintain standing position on its own following recovery. The difference between the two was taken as the duration of recumbency.

#### 2.6.4. Standing Time

Standing time was calculated by determining the difference between the time when the goat assumed sternal posture as sign of recovery and when the goat finally stood on its own unassisted.

#### 2.6.5. Quality of Induction and Recovery

The quality of induction and recovery were evaluated using a modified method according to Prassinos et al. [9]. Hence, “good,” “fair,” and “poor” were assigned to the experimental goats in each group according to the criteria depending on the observed signs.

#### 2.6.6. Induction Quality Scoring

*Good*. Smooth induction, rapidly assumed recumbency, no signs of excitement.

*Fair*. Slightly prolonged induction, mild excitation, presence of swallowing reflex.

*Poor*. Obvious excitement, jumps or attempts to stand after recumbency, full presence of swallowing reflex.

#### 2.6.7. Recovery Quality Scoring

*Good*. Smooth, easy transition to alertness, resumption of sternal position, ability to stand within a reasonable amount of time and ability to walk with minimal ataxia. 

*Fair*. Transient excitement or whole body movement, some struggles, hyperresponsiveness that disappears once the goat stands unassisted but with moderate ataxia.

*Poor*. Stereotype behaviour, for example, circling, premature attempts to stand, prolonged struggling.

### 2.7. Side Effects

Side effects like apnoea, laboured breathing and snoring, grunting, coughing, salivation, paddling movement, opened eye lid, ataxia, and urination were observed by watching the goats closely following induction of the anaesthesia, throughout the anaesthetic and postanaesthetic period.

### 2.8. Data Analysis

Data were expressed as mean ± standard error of mean in each group. The means were compared using analysis of variance (ANOVA). The means were separated using least significant difference (LSD). A probability value less than or equal to 0.05 (*P* ≤ 0.05) was considered statistical where applicable.

## 3. Result

### 3.1. Physiological Variables

**Figure 1:**
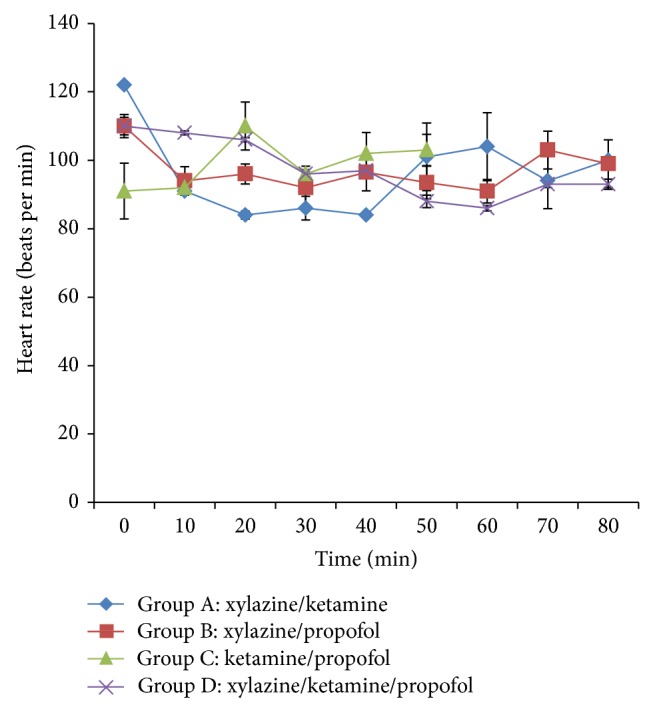
Mean heart rate following intravenous multimodal therapy of propofol, xylazine, and ketamine anaesthetics.

The heart rate significantly (*P* < 0.05) decreased in groups A, B, and D following induction when compared to group C. The heart rate was significantly (*P* < 0.05) lower in group A from 20 min to 40 min after induction when compared to other groups. However, the heart rate was significantly (*P* < 0.05) decreased in groups B and D at 60 min when compared to the control (group A). On the contrary, the heart rate significantly (*P* < 0.05) increased at 20 min after induction in group C (Figure 1).

**Figure 2:**
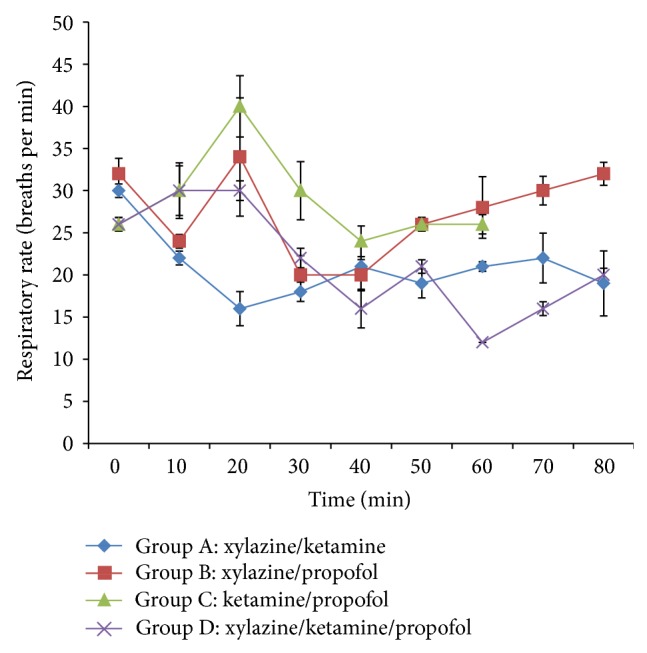
Mean respiratory rate following intravenous multimodal therapy of propofol, xylazine, and ketamine anaesthetics.

The respiratory rate decreased significantly (*P* < 0.05) in groups A and B at 10 min after induction However, it significantly (*P* < 0.05) increased in groups B, C, and D at 20 min after induction when compared to group A. The multimodal therapy also significantly (*P* < 0.05) decreased the respiratory rate in group D at 60 min after induction when compared to control (group A) (Figure 2).

**Figure 3:**
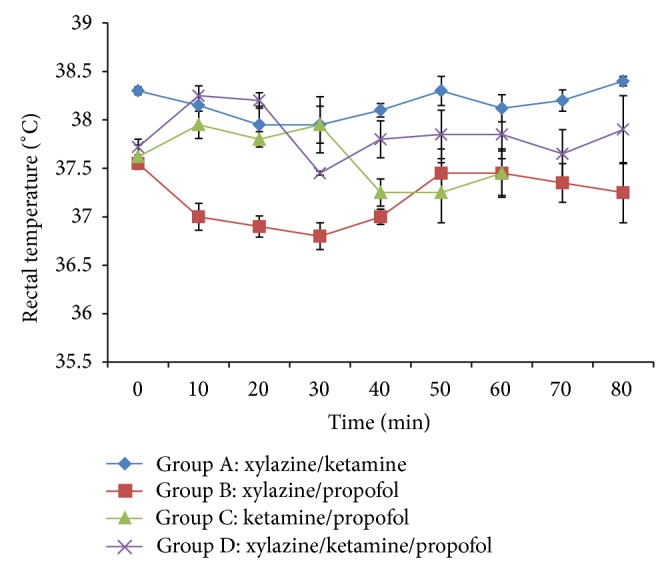
Mean rectal temperature following intravenous multimodal therapy of propofol, xylazine, and ketamine anaesthetics.

There was significant (*P* < 0.05) decrease in rectal temperature in group B from 10 min to 40 min after induction when compared to other groups. The temperature also significantly (*P* < 0.05) decreased in groups B, C, and D at 40 min when compared to group A. However, rectal temperature increased significantly in group D at 20 min after induction when compared to group A (Figure 3).

### 3.2. Anaesthetic Indices

The onset of analgesia was 3.57 ± 0.28 min, 2.15 ± 0.10 min, 1.80 ± 0.21 min, and 1.00 ± 0.00 in groups A, B, C, and D, respectively. The onset of analgesia in group D was significantly (*P* < 0.05) shorter when compared to the control and to other groups.

The period of surgical anaesthesia lasted for 51.00 ± 11.01 min, 51.50 ± 5.92 min, 40.30 ± 6.10 min, and 78.50 ± 1.04 min in groups A, B, C, and D, respectively. The surgical anaesthesia time was longer in group D and shorter in group C when compared the control group.

The mean recumbent time following induction was 91.50 ± 3.75 min, 65.04 ± 6.45 min, 45.02 ± 5.24 min, and 91.00 ± 2.08 min in groups A, B, C, and D, respectively. There is no significant (*P* > 0.05) variation in recumbent time between group D and control. However, recumbent time in groups B and C was significantly (*P* < 0.05) shorter when compared to the control.

The mean standing time was 7.50 ± 0.50 min, 4.58 ± 1.20 min, 1.30 ± 0.20 min, and 1.50 ± 0.40 min for groups A, B, C, and D, respectively. The mean standing time in groups B, C, and D was significantly (*P* < 0.05) shorter when compared to control groups.

### 3.3. Nature of Induction and Recovery

Following the administration of anaesthetics, smooth induction was observed in all the goats in groups B and D and three goats each in groups A and C. The goats in groups B and D rapidly assumed lateral recumbent position and no forms of excitement were observed. However, one goat each in groups A and C exhibited slight excitement and the presence of swallowing reflex was observed (Table 1).

Recovery was also smooth and the goats stood some minutes after assuming sternal recumbency. These were observed in all the goats in group D and three goats each in groups B and C. However, transient excitement, struggle to stand, and moderate ataxia were observed in all the goats in group A, one goat in group B, and two goats in groups C (Table 1).

### 3.4. Side Effects

The side effects observed in this experiment include apnoea, laboured breathing, snoring, grunting, coughing, salivation, tail wagging, paddling movement, open eye lid, ataxia, and urination.

Salivation was profuse in all the goats in groups A and B and two goats in group C, but mild in two goats in group D. Coughing was observed in one goat in group A only and one to three goats in groups A, B, and C. Ataxia was noticed in goats after standing in groups A and C, while urination was observed in all goats in group D and some goats in other groups. Snoring was present in groups B and C and highly present in group D. Open eyelids were observed in all goats in group A and two goats in group C. This persisted in group A even after maintenance with ketamine, but the eyes were closed following maintenance with propofol in group C (Table 2).

## 4. Discussion

Based on the common drug in use in clinical practice and in this breed of goat, group A (ketamine + xylazine) was used as the control in this experiment. The decrease in heart rate after induction in groups A, B, and D may be attributed to the bradycardia effect of the therapy used in these groups. Similar results have been reported following ketamine + xylazine anaesthesia [6, 14, 16, 17]. The increase in heart rate in group B may be as a result of light anaesthesia or as a result of cardiac stimulatory effect of ketamine [7].

The multimodal therapy used in the control (group A) decreased the respiratory rate more than the multimodal therapy used in other groups at induction. However, the profound decrease in respiratory rate in group D at 60 min may be attributed to the effect of maintenance dose. It means that ketamine + xylazine combination causes respiratory depression [18] and addition of propofol may lead to further decrease in respiratory rate especially at maintenance. However, oxygen and carbon dioxide tension which drive respiratory rate were not measured due to nonavailability of the monitoring equipment.

The decreased temperature recorded in group B is in accordance with the findings of Adetunji et al. [15]. However, the maintenance of anaesthesia did not prolong the drop in the rectal temperature beyond 40 minutes in all groups.

The synergistic effect of propofol and other drugs used in the therapy might have resulted in the short onset of analgesia in groups B, C, and D. Propofol causes rapid induction (within 5 to 30 sec) which results in unconsciousness [9].

Long surgical anaesthetic time was achieved in groups A, B, and D. This could be attributed to synergistic actions of the drugs used. Xylazine acts in synergism with ketamine to produce better analgesic effect [19].

The smooth induction recorded in most of the groups may also be attributed to the combined anaesthetic and sedative actions of the drugs used. However, rapid recovery may be attributed to noncumulative effect of propofol used for maintenance in groups other than the control.

Apnoea and salivation were the major side effects observed. The apnoea might have resulted from the depressive effect of the drugs on respiratory centre. Apnoea has been reported in goats after propofol administration [9] while ketamine alone or ketamine + xylazine caused apnoea in goat [9, 14, 16]. Salivation was seen in all groups but it was more profuse in groups A and C which have ketamine in their therapy. However, xylazine or ketamine + xylazine administration has been reported to cause salivation in goat under anaesthesia [14, 16, 20]. Bags were used to support the heads of the animal at the poll and saliva flew freely out of the mouth [19].

Urination was also observed in all the groups. However, it was more pronounced in groups B and D. Urination is a means of elimination of drugs by the kidney. It is a response of healthy kidney to eliminate these substances from the body. Urination following the administration of xylazine is a common occurrence in goat [20, 21]. Urination after ketamine-xylazine injection has been reported [14, 16].

## 5. Conclusion

Based on the results from this study, the multimodal intravenous therapy caused smooth and uneventful induction with mild cardiopulmonary depressions and rapid recovery.

Apnoea was the major side effect of the multimodal therapy more especially in propofol combinations. Therefore, care must be taken when administering these drugs by monitoring the animal closely to know when the apnoea develops. Once apnoea is noticed, drug administration should be paused until the animal regains normal regular breathing. An attempt to continue with the injection or infusion of drug once the apnoea develops may lead to respiratory arrest and possible death of the animal

Therefore, the multimodal therapy used in groups A, B, and D could be used successfully in both short and long surgical procedures without untoward side effects. However, the therapy used in group D could be the best combination therapy taking into account the parameters measured.

## Figures and Tables

**Table 1:** Nature of induction and recovery qualities (*n* = 4).

	Group A	Group B	Group C	Group D
Quality of induction	Good 75% Fair 25%	Good 100%	Good 75% Fair 25%	Good 100%
Quality of recovery	Fair 100%	Good 75% Fair 25%	Good 50% Fair 50%	Good 100%

% of animals that show respective signs in each goat group.

**Table 2:** Summary of the side effects observed in different experimental groups.

Side effects	Group A	Group B	Group C	Group D
Apnoea	+25%	+50%	+75%	+100%
Laboured breathing	+25%	+50%	−100%	−100%
Open eyelid	+100%	−100%	+50%	+25%
Snoring	−100%	+50%	+100%	+100%
Grunting	+50%	−100%	−100%	−100%
Coughing	+25%	−100%	−100%	−100%
Salivation	+100%	+50%	+100%	+50%
Tail wagging	+75%	−100%	+50%	−100%
Paddling movement	+75%	−100%	+75%	+50%
Ataxia	+75%	−100%	+100%	−100%
Urination	+25%	+75%	+50%	+100%

(−) Absence, (+) present, and (%) number of goats affected.
